# Michigan State Board of Health

**Published:** 1883-02

**Authors:** 


### ARTICLE V.

### MICHIGAN STATE BOARD OF HEALTH.

(Reported for the CHICAGO MEDICAL JOURNAL AND EXAMINER.)

The regular quarterly meeting of the Michigan State Board of Health was held at its office in Lansing, Michigan, on Tuesday, January 9, 1883, the following members being present: Hon. LeRoy Parker, of Flint, President; Henry F. Lyster, M.D., of Detroit; Rev. D. C. Jacokes, of Pontiac; J. H. Kellogg, M.D., of Battle Creek; Arthur Hazlewood, M.D., of Grand Rapids; John Avery, M.D., of Greenville, and Henry B. Baker, M.D., Secretary.

The subject of oil-inspection was brought up, and it was stated that it was alleged that much oil is being sold without being inspected, partly because of there not being enough inspectors, and partly because of the system of payment for inspection, which allows a man to receive large pay for his labor performed during the first of the month, and toward the last of the month, when the fees get smaller, the work is not done. The object to be attained by the inspection should be to ensure that the oil used by the remote farmer in his cottage, should be as safe to use as that in the city, near the inspector; and it was represented to the Board that the cost of inspection could be reduced from $27,000 to $12,000, and yet have all the oil properly inspected, but not without employing more inspectors than are now employed. Dr. Hazlewood and Dr. Baker were appointed a committee to take such action as was considered necessary on the subject of the inspection of oils.

The secretary made his report of work during the last quarter, mentioning the efforts to prevent the introduction of contagious diseases by immigrants; the distribution of blanks and circulars to officers of local boards of health, providing for their annual reports; the general distribution of the Annual Report of the Board for 1881; the issuing of a circular by Drs. Kellogg and Avery, with a view to collecting all facts respecting the cause and spread of diphtheria; the preparations for a sanitary convention at Pontiac, commencing January 31; the preparation of articles embracing facts collected respecting contagious diseases in Michigan; also one giving what has been learned by the immigrant-inspection service; also a paper read before a convention at Jackson, on "The Relations of the State Board of Health to Corrections and Charities." A resumè of the work of other State Boards of Health was also presented. It showed that in three counties in California, small-pox had been introduced by immigrant cars. The authorities provided an inspection, and have for the present stopped the introduction of this loathsome disease. In Michigan, the expense of small-pox inspection is much greater, on account of the large number of immigrants, and the secretary claimed that the national government should continue to provide means for such inspection, and thus protect the people from Maine to California. Senator Conger and Representative Rich have introduced in Congress bills to appropriate $25,000 to enable the National Board of Health to aid State and local boards of health to prevent the introduction of contagious diseases, and their spread from one State into another, by means of the immigrant-inspection service. After reading the above-mentioned bill, the following resolution was passed:

WHEREAS, This Board has long been laboring for the restriction and prevention of contagious diseases in Michigan, which depends greatly upon the existence of such diseases in other States and countries; and as this Board has been able to trace several outbreaks of disease in this State during the past year to immigrant travel, etc., therefore

*Resolved*, That this Board of Health urgently requests our members of Congress to endeavor to secure the passage of a bill to appropriate $25,000 for the remainder of this fiscal year, and thereafter at about the same rate, to enable the National Board of Health to coöperate with State and local boards of health and quarantines in efforts to prevent the introduction of contagious diseases into the United States, and their spread from one State to another.

The invitation to hold a sanitary convention at Reed City sometime in the spring was accepted.

Analyses of apple-butter, and of the tinned-copper, such as is used to make wash-boilers, were presented. The apple-butter is often made in such "copper" boilers when they are new. The acid of the fruit attacks the tin, which often contains lead in dangerous quantities, and it is said that the tin lining is eaten off in one or two times using for making apple-butter. The analysis of the apple-butter showed distinct traces of lead and tin, and a faint trace of copper. That portion of the apple-butter in contact with the cap of the fruit jar in which the apple-butter was sent to the chemist gave very strong reactions for zinc, doubtless derived from the zinc caps which screw down upon the mouths of "Mason" fruit jars. The specimen of tinned-sheet copper used in making "copper" boilers, and other kitchen utensils, was analyzed, and the "tin" was found to contain a large quantity of metallic lead; about two-fifths of the "tin" was lead. From one square foot of such tinned surface there was obtained the equivalent of 150 grains of metallic lead. The ordinary clothes-boiler, such as used in our kitchens, if made of this "tinned-copper," would have two and one-third ounces of metallic lead on its surface, an amount that must have a serious influence on persons who eat acid fruits and juices boiled in such a vessel. Dr. Hazlewood and Dr. Baker were requested to make an investigation into the subject of metallic poisoning by utensils for cooking and storing food. The city of Hillsdale, Michigan, now requires burial permits. While this subject was under discussion, Hon. Geo. Howell, M.D., a member of the Committee on Public Health, of the Present House of Representatives, read a proposed bill to promote the public health. He received the thanks of the Board. The subject of requiring burial permits and thus securing mortuary statistics before removal of the body of deceased persons, was referred to the Committee on Legislation, with the request to prepare a bill and submit it to the Legislature. The Board adopted a resolution commending the effort to secure mortuary statistics before the burial or removal of the body of a deceased person; and declaring that the method is applicable to townships and villages as well as to cities.

Many letters from prominent sanitarians in this and other countries were presented which spoke in a very appreciative manner of the work of this Board.

It was moved and carried that local boards of health be recommended to supply physicians and persons acting as such, with blank notices of diseases dangerous to the public health printed on postal eards.

The Committee on Legislation reported on an act requiring the registration of plumbers. Hon. James Heuston, M.D., Senator, spoke on the subject of the examination of new dwellings before occupancy, and the following resolution was offered by Dr. Baker and adopted:

*Resolved*, That the Committee on Legislation, etc., and the Committee on Buildings, Public, Private, etc., jointly, be requested to take into consideration the feasibility of the suggestion made at this meeting by Hon. James Heuston, M.D., for a State law requiring all plans for new dwellings to be submitted to the local Board of Healh for approval.

The Committee on Disposal of Excreta was requested to prepare a pamphlet for general distribution, on the disposal of slop-water, garbage, etc., in villages without a sewerage system, and from detached dwellings.

The American Public Health Association has recommended making it a penal offense to communicate a contagious disease. The Committee on Legislation was requested to modify the bill so as to name diphtheria, scarlet fever, and small-pox, and get the subject before the Legislature.

The Special Committee on Sanitary Conventions reported that a Sanitary convention was to be held at Pontiac on January 31 and February 1, 1883. Among the subjects to be presented, are papers on the Limitation and Prevention of Typhoid Fever, the Relation of the Medical Profession to Public Health Laws, on Toy Pistols, the Dangers in Dirt, and on the Contamination of Well Water.

The following resolutions (two of them having been passed before) were reaffirmed:

*Resolved*, That there should be required of all who are to begin the practice of medicine in this State an examination as to their qualifications.

*Resolved*, That such examinations by the State should be restricted to questions in demonstrable knowledge as distinguished from questions of mere opinion.

*Resolved*, That, as a public health measure these two resolutions be referred to the President and Secretary, with a request to do what they can to further the objects of the resolutions.

Auditing of bills and other routine business was performed. The next meeting of the Board will be at Pontiac, January 31, and the next regular meeting will be April 10, 1883.

---

DR. THOMAS WATSON died December 12, 1882. He was in his ninety-second year. He is perhaps best known for his classical work on the Practice of Medicine. It is not too much to say that no other work of similar extent upon this subject has ever so charmed every reader. On Friday, the 15th instant, the remains were conveyed in a plain coffin of polished oak, without other covering than a floral cross, from the residence of his son to the burial-ground attached to the Reigate parish church. At the entrance of the churchyard the procession was met by the clergy and a surpliced choir, who chanted the opening sentences of the service. The greater part of the service, including the singing of the two well known hymns, "Lead Kindly Light," and "Ten Thousand Times Ten Thousand," was performed within the church. There was afterward a procession to the grave, the bare earth of which had been concealed from view by a lining of evergreens and flowers. Among those forming the procession were the son and heir of the deceased, Sir Arthur Watson, Lady Watson, Miss Watson, the two sons and two daughters of Sir Arthur and Lady Watson, and Captain Watson, a nephew of Sir Thomas. The medical attendants of the deceased were all present—namely, Dr. Walters, Dr. George Johnson, Mr. Lister, Dr. Greenhow, and Dr. Holman. The College of Surgeons was represented by the President, Mr. Spencer Wells, and the College of Physicians by the Registrar, Dr. Pitman. Sir William Jenner wrote to express his regret that he was unavoidably prevented from attending.

